# Cyclodextrin-containing hydrogels as an intraocular lens for sustained drug release

**DOI:** 10.1371/journal.pone.0189778

**Published:** 2017-12-15

**Authors:** Xiao Li, Yang Zhao, Kaijie Wang, Lei Wang, Xiaohui Yang, Siquan Zhu

**Affiliations:** 1 Beijing Tongren Eye Center, Beijing Key Laboratory of Ophthalmology and Visual Science, Beijing Tongren Hospital, Capital Medical University, Beijing, China; 2 CAS Center for Excellence in Nanoscience, CAS Key Laboratory for Biological Effects of Nanomaterials and Nanosafety, National Center for Nanoscience and Technology (NCNST), Beijing, China; 3 Beijing Institute of Ophthalmology, Beijing Tongren Hospital, Capital Medical University, Beijing, China; Chung-Ang University College of Engineering, REPUBLIC OF KOREA

## Abstract

To improve the efficacy of anti-inflammatory factors in patients who undergo cataract surgery, poly(2-hydroxyethyl methacrylate-co-methyl methacrylate) (p(HEMA-co-MMA)) hydrogels containing β-cyclodextrin (β-CD) (pHEMA/MMA/β-CD) were designed and prepared as intraocular lens (IOLs) biomaterials that could be loaded with and achieve the sustained release of dexamethasone. A series of pHEMA/MMA/β-CD copolymers containing different ratios of β-CD (range, 2.77 to 10.24 wt.%) were obtained using thermal polymerization. The polymers had high transmittance at visible wavelengths and good biocompatibility with mouse connective tissue fibroblasts. Drug loading and release studies demonstrated that introducing β-CD into hydrogels increased loading efficiency and achieved the sustained release of the drug. Administering β-CD via hydrogels increased the equilibrium swelling ratio, elastic modulus and tensile strength. In addition, β-CD increased the hydrophilicity of the hydrogels, resulting in a lower water contact angle and higher cellular adhesion to the hydrogels. In summary, pHEMA/MMA/β-CD hydrogels show great potential as IOL biomaterials that are capable of maintaining the sustained release of anti-inflammatory drugs after cataract surgery.

## Introduction

A cataracts is defined as an opacification on the ocular lens. Cataracts have become the leading cause of blindness worldwide because an extended life expectancy has resulted in an aging population [1,2]. Both the incidence and the prevalence of cataracts significantly increase with age. Currently, a strategy using phacoemulsification to extract the opaque lens followed by the subsequent implantation of an intraocular lens is considered the best method to restore vision [3]. However, post-operative complications, such as inflammation, infection, and posterior capsule opacification (PCO), can occur after cataract surgery and threaten the recovery of the patient’s vision [4–6]. Inflammation is one of the most common problems in affected patients [7]. Both cataract surgery (considered a traumatic stimulation) and coexisting ocular diseases (such as uveitis) can promote the migration of inflammatory cells and the release of inflammatory factors, resulting in post-operative inflammation [8]. Corticosteroids, such as dexamethasone and hydrocortisone, are widely used to prevent inflammation post-cataract surgery [9,10]. Ophthalmic formulations, such as dexamethasone eye drops, are the primary mode of topical administration [11]. However, these formulations have several drawbacks, such as low bioavailability (below 5.0%) and an inability to control the concentration of the drug in the aqueous humor [12,13]. Moreover, frequently administering eye drops may result in the poor compliance of patients, especially older and infant patients [14]. A number of drug delivery strategies have been developed to overcome the limitations of eye drops. For instance, sustained release capsules containing dexamethasone were embedded in the aqueous humor and maintained a preferable drug concentration, although the argument over the toxicity of the products of capsule degradation is not yet resolved [15]. In addition, *in vitro* experiments showed that the drug sustained the release properties of contact lenses. However, both the tear film and the cornea can hinder the migration of a drug from an extra-ocular contact lens into the eye or dilute the drug’s concentration [16].

We sought to design and prepare a self-anti-inflammatory IOL that could be pre-operatively loaded with corticosteroids and used to post-operatively release the drug [17]. Hydrophilic materials, such as p(HEMA-co-MMA) copolymers, are widely used as IOLs because of their excellent biocompatibility, high rate of transmission and thermal stability [18]. In particular, the relative hydrophilicity of these copolymers allows them to achieve high permeability in aqueous media, meaning that the copolymers themselves are effective drug carriers [19]. However, the major disadvantages of using p(HEMA-co-MMA) copolymers as a drug delivery system are that they can be loaded with only a low amount of the drug and they have short drug-release times [20]. Several method have been developed to achieve long-term drug release from IOLs. These include attaching IOLs to a drug delivery accessory, depositing a coating of the drug on the IOL surface, and inducing the copolymerization of IOL monomers and modified drugs [21–25]. Cyclodextrins (CDs) are a group of macromolecules with a hydrophobic cavity. CDs have been extensively used as drug carriers for hydrophobic drug molecules [26–28]. Moreover, CDs have also been demonstrated to enhance the drug release capabilities of hydrogels by forming inclusion complexes with various small drug molecules [29].

In recent years, our group has developed several functional IOL materials, such as injectable IOL materials and shape-memory IOL materials [30–32]. In this attempt to apply anti-inflammatory agents following cataract surgery, we reported self-anti-inflammatory IOLs that were based on pHEMA/MMA/β-CD hydrogels and demonstrated the capability of sustained dexamethasone release (S1 Fig). These IOL biomaterials were systematically evaluated *in vitro* to determine their properties, including their optical and thermomechanical properties, and their equilibrium swelling ratio (ESR), cytotoxicity, and dexamethasone loading and release behaviors. Because surface characteristics play important roles in the biocompatibility of IOLs, we also used the surface wettability and lens epithelial cell (LEC) adhesion test to evaluate pHEMA/MMA/β-CD hydrogels. The results of these experiments indicated that the pHEMA/MMA/β-CD hydrogels were suitable IOLs materials for the *in situ* drug release of anti-inflammatory agents after cataract surgery.

## Materials and methods

The 2-HEMA, MMA, ethylene glycol dimethacrylate (EGDMA), 3-methylbenzoic acid (3-MBA) and β-CD were purchased from Sigma-Aldrich, Beijing. The 2,2’-azobisisobutyronitrile (AIBN), acryloyl chloride and other solvents were obtained from J&K Scientific, LTD. All reagents and solvents were used without further purification.

### Synthesis of triple-methacrylated-β-CD (Triple-MA-β-CD)

As described in the literature, triple-MA-β-CD was synthesized using a traditional method based on acryloyl chloride (S1 Fig) [33]. In detail, 6.0 g β-CD (previously dried at 100.0°C for 12 h) was dissolved in a mixture of 40.0 mL of dried N,N-dimethylformamide (DMF) and 14.0 mL of dried triethylamine (TEA) while magnetically stirred for more than 20 min. Then, 5.0 mL of acryloyl chloride was slowly dropped into the mixture at 0°C while in an atmosphere of nitrogen. The resulting mixture was stirred for another 30 min at room temperature and then vacuum-filtrated. Then, acetone was added to the filtrate drop by drop to obtain a precipitation product. The solvent was removed, and the precipitate was washed with acetone at least three times to further purify the product. Finally, the precipitate was collected and dried at room temperature under a vacuum. The ^1^H NMR spectra of β-CD was then recorded using a Bruker AMX400 apparatus (Germany).

### Preparation of pHEMA/MMA/β-CD hydrogels

MMA, EGDMA and Triple-MA-β-CD were dissolved in HEMA using the formulation listed in Table 1. After AIBN was added, the mixture was immediately injected into molds made using two glass plates coated internally with a polypropylene film. The molds were separated by a silicon frame (1 mm thick) [34]. Then, the molds were placed in an oven at 50.0°C for 12 h and then heated to 70.0°C for another 24 h. After polymerization, the gel plates were immersed in boiling water for 15 min to extract the unreacted monomers. Each gel was then cut into disks (10.0 mm in diameter), and the disks were hydrated in distilled water for 3 days. The water was replaced every 12 h. The disks were then dried in an oven at 105.0°C for 24 h to obtain the disk-shaped samples that were used to evaluate drug loading and *in vitro* release profiles.

**Table 1:** Formulations of pHEMA/MMA/β-CD hydrogels.

Samples	Monomers (wt.% of total monomers)		Drug carrier (wt.% of total monomers)	Crosslinker (wt.% of total monomers)	Initiator (g)
	HEMA	MMA	Triple-MA-β-CD	EGDMA	AIBA
pHEMA/MMA	85.58	14.00	—	0.42	0.0075
pHEMA/MMA/β-CD1	83.21	13.61	2.77	0.41	0.0075
pHEMA/MMA/β-CD2	80.96	13.24	5.40	0.40	0.0075
pHEMA/MMA/β-CD3	78.84	12.90	7.88	0.39	0.0075
pHEMA/MMA/β-CD4	76.82	12.57	10.24	0.38	0.0075

### Characterization of pHEMA/MMA/β-CD hydrogels

#### Weight loss ratio before and after extraction

The pre-extraction weight of the dried samples [M_1_ (g)] was measured before they were immersed in boiling water. The post-extraction weight of the samples [M_2_ (g)] was determined after they were dried at 105.0°C. The weight loss ratio (wt.%) of pHEMA/MMA/β-CD hydrogels was calculated as follows:

$$\text{Weight}\;\text{loss}\;\text{ratio}\left(\%\right)=\frac{\left({\text{M}}_{1}-{\text{M}}_{\text{2}}\right)}{{\text{M}}_{1}}\times \text{100\%}$$ (1)

#### Fourier-transform infrared (FT-IR) spectroscopy analysis

The FT-IR spectra of the prepared pHEMA/MMA/β-CD hydrogels were recorded over the range 400.0–4000.0 cm^-1^ using a FT-IR spectrophotometer (Perkin-Elmer) with the potassium bromide pellet technique. The FT-IR spectra of the unmodified β-CD and Triple-MA-β-CD were evaluated using the same methods.

#### Thermo-mechanical properties

The glass transition temperature (T_*g*_) of the pHEMA/MMA/β-CD hydrogels was measured using differential scanning calorimetry (DSC) with a DSC Q2000 (TA Instruments, New Castle DE, USA) equipped with a refrigerated cooling accessory. Nitrogen (flow rate: 50.0 mL min^-1^) was used to purge the gas. To determine the T_*g*_, 5.0–10.0 mg of the dried samples were accurately weighed and then placed in crimped aluminum pans and covered with a lid. All of the samples were run using alumina as reference at temperatures ranging from 40.0°C to 200.0°C (the temperature was raised 20.0°C min^-1^). Mechanical properties were evaluated using standard dumbbell-shaped specimens that were cut from prepared wet gel plates using a dumbbell-shaped cutting knife (test section size: 10.0 mm × 40.0 mm). A Series IX Automated Materials Testing System (Instron Corp, USA) was used to determine the elastic modulus, tensile strength and elongation at break of the samples at a stretching rate of 1.0 mm sec^-1^ at 37.0°C and a relative humidity of 60.0%.

#### Optical properties

The transparency of the pHEMA/MMA/β-CD hydrogels was measured at 300.0–800.0 nm using a Shimadzu 2600 UV/vis spectrophotometer. Its refractive index (RI) was determined using an Abbe refractometer (WAY, Shanghai Precision & Scientific Instrument, China).

#### ESR

Dried, disk-shaped samples were immersed in distilled water for 24 h until they achieved swelling equilibrium. The weights of the wet samples [M_w_ (g)] were determined after moisture was carefully cleaned from the surface using a soft tissue. The weights of the dried samples [M_d_ (g)] were measured after they were dried at 105.0°C for 24 h. The ESR of the pHEMA/MMA/β-CD hydrogels was calculated using the following equation:

$$\text{ESR}\left(\%\right)=\frac{\left({\text{M}}_{\text{w}}-{\text{M}}_{\text{d}}\right)}{{\text{M}}_{\text{w}}}\times \text{100}\%$$ (2)

#### Surface wettability

The water contact angles (CAs) of the wet samples were determined using an OCA15 contact angle analyzer (Dataphysics, Germany). Drops of distilled water (~1.0 μL in volume) were applied to the surfaces of the samples.

#### Functional β-CD content

Dried pHEMA/MMA/β-CD disks were soaked in 10.0 mL of 3-MBA aqueous solution (0.5 mg mL^-1^) at 37.0°C for 48 h in the dark. The concentration of 3-MBA was determined at 281.0 nm using a Shimadzu 2600 UV/vis spectrometer. The total amount of 3-MBA loaded in each of the samples was defined as the difference between the amount in the solution before and after immersion. The proportion of functional β-CD in the samples was estimated by determining the amount of 3-MBA that specifically combined with single β-CD in the hydrogels.

#### Drug loading and release

Dried pHEMA/MMA/β-CD disks were immersed in 10.0 mL of a 0.04 or 0.08 mg mL^-1^ dexamethasone aqueous solution (in Milli-Q water) at 25.0°C to load the dexamethasone. In addition, in some systems, samples were immersed in a 0.08 mg mL^-1^ dexamethasone solution and placed in an oven at 37.0°C to determine whether heat influenced loading efficiency. In all of the above systems, the samples were incubated in the dark for 48 h to achieve absorption equilibrium. The amount of dexamethasone that was loaded in the hydrogels was defined as the difference between the initial amount of dexamethasone in the aqueous solution and the amount that remained after loading. The samples were analyzed at 240.0 nm using a Shimadzu 2600 UV/vis spectrometer.

Samples loaded with dexamethasone were rinsed with distilled water and then placed in 4.0 mL of distilled water at 37.0°C. A 2.0 mL sample solution was periodically obtained from the immersion medium, which was supplemented with the same volume of pure distilled water. The amount of dexamethasone released by the samples was determined using a spectrophotometer at 240.0 nm.

#### Cytotoxicity

L929 cells (mouse connective tissue fibroblasts, obtained from the Chinese Academy of Preventive Medical Sciences, Beijing, China) were maintained in Dulbecco’s modified Eagle’s medium (DMEM; HyClone) supplemented with 10.0 vol.% heat-inactivated fetal bovine serum (FBS, HyClone), penicillin (100 U mL^-1^) and streptomycin (100 μg mL^-1^) in a humidified incubator in 5.0% CO_2_ at 37.0°C [35]. The cells were cultured to the end of logarithmic growth phase when 80.0% of the cells showed a tendency to fuse. In subsequent cultures, the cell monolayer was washed twice using phosphate-buffered saline (PBS) and then incubated with trypsin-EDTA cell dissociation buffer (0.05% trypsin and 0.25% EDTA) for 5 min at 37.0°C to achieve separation. Then, the L929 cells were suspended in culture medium, and the cell suspension was diluted to a cell density of 2.0 × 10^4^ mL^-1^. Samples of the resulting L929 cell suspension (500 μL) were seeded in 24-well tissue culture plates (Costar) and co-cultured with the sterilized pHEMA/MMA/β-CD disks in DMEM for 7 days. The medium was replaced every 2 to 3 days. Samples that had been previously autoclaved were then immersed in PBS for 48 h under sterile conditions. The cultures were analyzed using inverted phase contrast microscopy to determine cell morphology on the 1^st^, 2^nd^, 4^th^ and 7^th^ day. Cell viability and proliferation were evaluated using MTT assays [36]. In addition, toxic organotin polyvinyl chloride (PVC) and high-density polyethylene (HDPE) films were used as the positive and negative controls in the cytotoxicity tests.

#### LEC adhesion tests

SRA01/04 cells (a human LEC line, obtained from the Chinese Academy of Preventive Medical Sciences, Beijing, China) were used to assess the lens capsule biocompatibility of pHEMA/MMA/β-CD hydrogels [37]. The culture and detachment procedures used in experiments involving SRA01/04 cells were similar to those used in L929 cells except for the amount of FBS (15.0 vol.%). Cell density was adjusted to 2.0 × 10^5^ mL^-1^. Then, 100.0 μL of the cell suspension was seeded on the bottom of 96-well plates, which were then embedded with sterilized disk-shaped samples (6.0 mm in diameter). The samples and cell suspensions were then co-cultured for 24 h. The morphologies of the SRA01/04 cells were evaluated using inverted phase contrast microscopy, and the amounts of SRA01/04 cells that had adhered to the surfaces of the samples were detected using crystal violet assays [38].

### Data processing method

All of the qualitative tests were performed in duplicate, and the secondary results were adopted. The quantitative experiments performed in this study were each repeated at least 3 times. The results were expressed as the mean ± standard error of mean (SEM) and compared using one-way ANOVA and student’s *t*-test. The level of statistical significance was set at 5% (P<0.05).

## Results and discussion

### Synthesis of triple-MA-β-CD monomers

**Fig 1:**
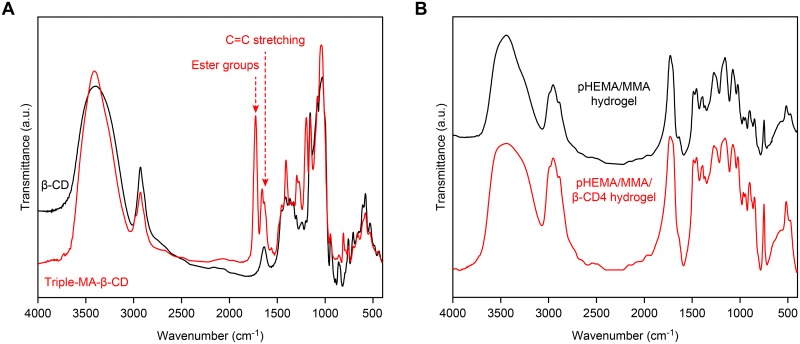
FT-IR spectra. (A) β-CD and Triple-MA-β-CD; (B) dried hydrogels: pHEMA/MMA hydrogel and pHEMA/MMA/β-CD3 hydrogel.

Fig 1A shows the FT-IR spectra of β-CD and Triple-MA-β-CD. The appearance of a peak at 1726 cm^-1^ (responsible for the ester groups) and an increase in absorbance at 1658 cm^-1^ (characteristic of C = C double bond stretching) confirmed the formation of Triple-MA-β-CD. The H NMR spectra (^1^H NMR (D_2_O, 400.0 MHz): δ 6.61–5.96 (9H, m), 5.27–5.05 (7H, m), 4.08–3.76 (28H, m), and 3.75–3.49 (14H, m)) indicated that the degree of substitution of the 21 hydroxyl groups on β-CD by methacrylic groups was 3. Assuming that Triple-MA-β-CD has a molecular weight of 1297.0 Da, the yield of the procedure was approximately 76.3 ± 4.8%.

### Preparation of pHEMA/MMA/β-CD hydrogels

Acrylates and methacrylates are commonly used as IOL biomaterials because they have a high RI and favorable biocompatibility and are FDA-approved. They were therefore chosen as the basic monomers with which to synthesize p(HEMA-co-MMA) copolymers [39]. In particular, HMEA and MMA were selected because of their relatively high hydrophilicity, which is associated with a preferable amount of drug-loading when using small molecular drugs [40]. As shown for the formulations listed in Table 1, pHEMA/MMA/β-CD hydrogels were prepared using radical copolymerization without solvents during processing. Unlike unsubstituted β-CD, the solubility of Triple-MA-β-CD was superior to the HEMA/MMA mixture. After polymerization, the hydrogels were immersed in boiling water to extract any unreacted monomers. The post-extraction weight loss ratio of the copolymers was below 1.0% (S2 Fig), and this meets the FDA extraction standard for IOLs. In addition, the disk-shaped samples were accurately weighed. We found that as the proportion of β-CD increased, the weight of dried samples that had nearly the same size (10.0 mm in diameter and 1.0 mm in thickness) decreased. This effect was attributed to the presence of hydrophobic cavities in the β-CD molecules (S3 Fig).

Fig 1B shows the FT-IR spectra of the dried pHEMA/MMA and pHEMA/MMA/β-CD hydrogels. The FT-IR spectra of the samples were similar before and after Triple-MA-β-CD was incorporated. Mainly, there were hydroxyl groups at 3700–3050 cm^-1^, the ester groups and carbonyl groups of pHEMA/MMA/β-CD were at 1730 cm^-1^, and the peak at approximately 1217–1110 cm^-1^ was assigned to the ether groups of β-CD and the pHEMA/MMA component of the hydrogels.

### Thermo-mechanical properties

The T_*g*_ and elastic modulus are two parameters that are very important for IOL biomaterials. On the one hand, implanting materials with a T_*g*_ below 37.0°C into the eyes may induce instability in the molecular chains, making them unsuitable for IOLs. On the other hand, the IOLs are deformed by the process of folding and preassembling them into injectors before implantation, and these deformations are associated with the elastic modulus of the IOL biomaterials.

**Fig 2:**
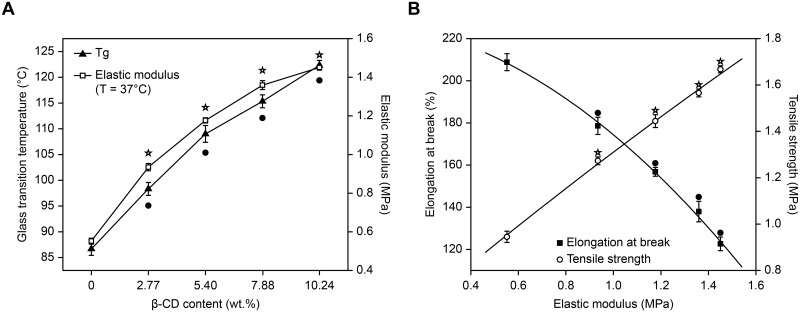
Thermo-mechanical properties. (A) T_*g*_ (● P<0.05 vs control) and elastic modulus (☆ P<0.05 vs control) of each of the pHEMA/MMA/β-CD hydrogels as a function of β-CD content. (B) Elongation at break (● P<0.05 vs control) and tensile strength (☆ P<0.05 vs control) at 37°C relative to the elastic modulus of the pHEMA/MMA/β-CD hydrogels.

Fig 2A shows the T_*g*_ and elastic modulus of the pHEMA/MMA/β-CD hydrogels. According to a DSC thermograph, the control pHEMA/MMA hydrogels underwent a glass to rubber transition at 86.67°C, which is similar to the values obtained in a previous report on pHEMA/MMA networks [41]. The incorporation of Triple-MA-β-CD into the hydrogels significantly increased the T_*g*_ of the samples. Moreover, there was a positive correlation between T_*g*_ (range from 86.67°C to 122.33°C) and the β-CD content. This result was attributed to an increase in cross-linking points, which was accompanied by a decrease in average molecular weight between the cross-linked points following the incorporation of Triple-MA-β-CD [42]. The elastic modulus of the pHEMA/MMA/β-CD hydrogels was measured using mechanical tensile testing at 37.0°C to simulate *in vivo* conditions. Dried samples exhibited increased fragility, and we therefore measured the elastic modulus of wet samples. As shown in Fig 2A, the elastic modulus steeply and nonlinearly increased as the concentration of β-CD increased. This was mainly because the incorporation of the rigid β-CD molecule, which has a large volume, decreased the flexibility of the chain structure of the copolymers [43]. The tensile strength and elongation at break of each pHEMA/MMA/β-CD hydrogel relative to its elastic modulus is shown in Fig 2B. As the elastic modulus increased, the tensile strength of the samples also increased, whereas elongation at break decreased. Thus, there was an inherent trade-off between elongation at break and tensile strength. In recent years, cataract surgery has become a minimally invasive refractive surgery, meaning that bigger IOLs can be implanted through smaller incisions. Therefore, in IOL biomaterials, a higher elongation at break is much more important than a higher tensile strength because the eyeball originates only a small amount of stress. Nevertheless, in this study, the elongation at break of the pHEMA/MMA/β-CD4 hydrogel with the highest β-CD content (10.24 wt.%) was still over 100.0%, making it suitable as an IOL biomaterial.

### Optical properties

**Fig 3:**
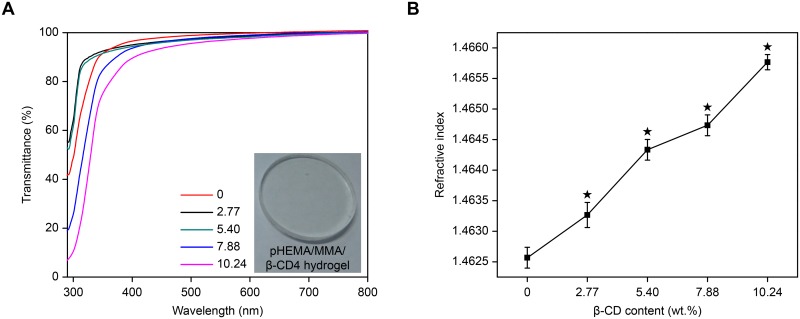
Optical properties. (A) Spectral transmittance of the pHEMA/MMA/β-CD hydrogels. (B) Refractive index of the pHEMA/MMA/β-CD hydrogels as a function of β-CD content (★ P<0.05 vs control).

In the eyes, IOLs act in a manner similar to a camera lens. Hence, an IOL can regulate the intensity and focus of light that enters the eyes, and the optical properties of IOL biomaterials are therefore especially important. In this study, transmittance and RI were used to evaluate the optical properties of pHEMA/MMA/β-CD hydrogels. Fig 3A shows the spectral transmittance of the samples. All of the samples had a transparent appearance and a high spectral transmittance (≥80.0%) at visible wavelengths (400.0–800.0 nm). In particular, each sample had a higher transmittance (>90.0%) at 600.0 nm, indicating that the hydrogels have excellent optical properties.

Fig 3B shows the RI values of the samples, which were originally approximately 1.464 and then increased slightly from 1.463 to 1.466 as β-CD content increased. The high RI of the hydrogels results from the high RI of HEMA and MMA because materials that contain chain-like structures always have a high RI. Moreover, the incorporation of Triple-MA-β-CD enhanced these chain-like structures. The RI of commercial hydrophilic acrylic IOLs is approximately 1.46 ± 0.3 [44]. In addition, IOLs that consist of materials with a higher RI can be much thinner, which would be helpful for reducing the size of surgical incisions, especially during minimally invasive cataract surgery [45].

### ESR

**Fig 4:**
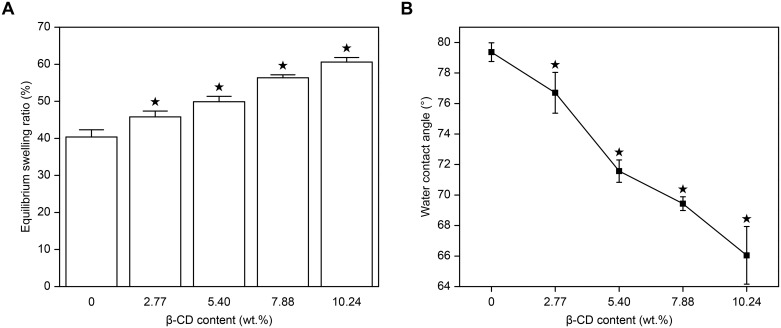
ESR (A) and water CAs (B) of pHEMA/MMA/β-CD hydrogels (★ P<0.05 vs control).

The concentration of water in a hydrophilic IOLs biomaterial is closely related to its hydrophilicity, surface wettability and drug-loading capacity. Here, ESR was used to evaluate the concentration of water in the pHEMA/MMA/β-CD hydrogels. Fig 4A shows the relationship between β-CD content and ESR. The control group (pHEMA/MMA hydrogels) had an ESR of 40.37%, which is similar to previously reported values [46]. As β-CD content increased, the ESR of the samples increased from 40.37% to 60.60%, probably because of the hydrogen bonds that formed between the unmodified hydroxyl groups in Triple-MA-β-CD and water molecules.

### Surface wettability and LEC adhesion tests

In modern cataract surgeries, a high incidence of PCO remains a concern [47,48]. The results of recent studies have indicated that the remnant space between an IOL and the lens posterior capsule plays an essential role in the occurrence of PCO because it indicates that the lens capsule is not completely filled by the IOL [49]. Linnola et al. proposed the “Sandwich theory” to explain the mechanism underlying PCO [50]. Briefly, if an IOL is likely to adhere to LECs, the remnant space can be filled with a monolayer of LECs, and this will prevent adhesion by extra LECs. The surface of the IOL, the LEC monolayer and the lens posterior capsule consequently form a sandwich-like structure that prevents the occurrence of PCO. This method is also described as “no space, no PCO”.

**Fig 5:**
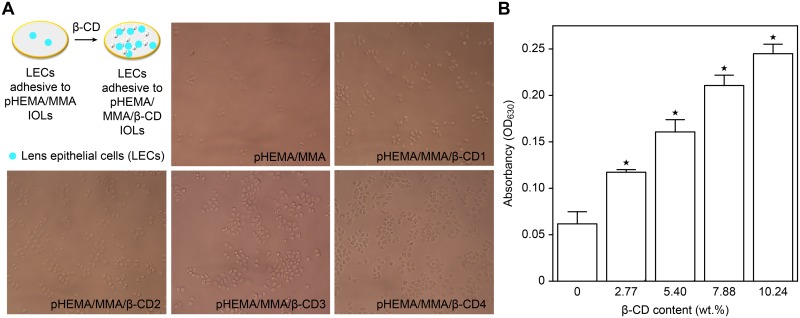
LEC adhesion test. (A) Inverted phase contrast microscopy of LECs that had adhered to wet samples. (B) The absorbency of LECs that had adhered to samples (shown at 630.0 nm) as a function of β-CD content (★ P<0.05 vs control). All microscopy images are at the same magnification (40 ×).

Previous studies have demonstrated that the capacity of LECs to adhere to an IOL surface can be evaluated by measuring the surface CA [51]. As shown in Fig 4B, the water CAs in the pHEMA/MMA/β-CD hydrogels ranged from 79.37° to 66.05° and decreased as the β-CD content increased. Furthermore, LEC adhesive tests were carried out to confirm the relationship between the surface wettability of the hydrogels and their ability to adhere LECs. As shown in Fig 5A, the amount of LECs that adhered to the control pHEMA/MMA hydrogels was smaller than the amount that adhered to the pHEMA/MMA/β-CD hydrogels that contained β-CD. The amount of LECs that adhered to the surfaces of the pHEMA/MMA/β-CD hydrogels increased as the β-CD content increased. All of the LECs spread well and exhibited a typical epithelial cell morphology. The amount of LECs was evaluated by measuring absorbency at 630.0 nm using a microplate reader (crystal violet staining, Fig 5B). There was a positive correlation between the absorbency of the LECs and β-CD content, and the observed difference was statistically significant. Based on the results described in this section, we conclude that the higher the β-CD content is, the lower the CAs of the samples are and the greater the amount of LECs that will adhere to the surface of the sample. Therefore, according to the “Sandwich theory”, the incorporation of Triple-β-CD into the pHEMA/MMA hydrogels may confer protection against PCO.

### Capability of functional β-CD in pHEMA/MMA/β-CD hydrogels

To determine the capability of small drug molecules to combine with pHEMA/MMA/β-CD, 3-MBA was used as a model drug, and its access to β-CD cavities was evaluated. This factors was used because it has a high affinity for β-CD [52]. Table 2 shows the amount of 3-MBA that was loaded into the samples (per gram) and the number of 3-MBA molecules that was loaded into a single β-CD cavity. The amount of 3-MBA that was taken up by the control pHEMA/MMA hydrogels was 20.93 mg g^-1^, and it was mainly found in the aqueous phase. As the β-CD content increased, the amount of 3-MBA that was loaded by the pHEMA/MMA/β-CD hydrogels markedly increased. The difference was statistically significant (P<0.05).

**Table 2:** The amount of 3-MBA loaded into each pHEMA/MMA/β-CD hydrogel.

Sample Code	3-MBA loaded into the samples	
	Total (mg g^-1^ in dried hydrogels)	Molar ratio (mol mol^-1^ β-CD)
pHEMA/MMA	20.93 (0.72)	-
pHEMA/MMA/β-CD1	22.51 (1.45)	0.54 (0.13)
pHEMA/MMA/β-CD2	25.62 (1.59)	0.93 (0.09)
pHEMA/MMA/β-CD3	28.41 (0.54)	1.04 (0.14)
pHEMA/MMA/β-CD4	32.63 (1.53)	1.24 (0.06)

In addition, 3-MBA can exist in pHEMA/MMA/β-CD hydrogels in the following states: dissolved in the aqueous phase of the wet hydrogel, absorbed non-specifically to the hydrogel backbone or specifically combined with β-CD. Thus, the number of 3-MBA molecules that can be combined in a single β-CD cavity can be defined as the difference between the total loading amount and the amount that exists in the aqueous phase. As presented in Table 2, there was a positive relationship between the β-CD content in the samples and the molar ratio of 3-MBA to β-CD. Interestingly, more than one 3-MBA molecule can be combined into one β-CD cavity when the proportion of β-CD in the hydrogels was equal to or more than 7.88 wt.%. It was previously demonstrated that 3-MBA molecules can form dimers (or structures with an even higher order) when suspended in a relatively apolar medium [53]. Therefore, some 3-MBA molecules that are loaded into the hydrogels while in the aqueous phase could form dimers with other molecules that were partially combined within the β-CD cavities.

As described above, the concentration of water in the hydrogels increased as β-CD content increased, whereas mesh size decreased as a result of an increased degree of polymerization. On the one hand, hydrogels with a higher water content load more drugs in the aqueous phase, causing them to release a greater initial burst of the drug. On the other hand, a smaller mesh size conferred a longer sustained release. This effect was observed in the dexamethasone release test described below.

### Drug loading

Dried pHEMA/MMA/β-CD disks were immersed in an aqueous solution of dexamethasone (0.08 mg mL^-1^) at 25.0°C to load the drug. The amount of drug that effectively loaded into the hydrogel was significantly affected by the concentration of the drug and the immersion temperature. Hence, in some system, the dexamethasone concentration was adjusted to 0.04 mg mL^-1^ or the immersion temperature was set to 37.0°C. Hydrogels that were loaded with dexamethasone reached equilibrium after 48 h of immersion.

Table 3 shows the amounts of dexamethasone that were loaded by the samples at equilibrium. More dexamethasone was loaded when either the dexamethasone concentration in the aqueous solution or the immersion temperature was higher (P<0.05). We estimated that ~15.28% and ~7.67% of the dexamethasone in the soaking solution was loaded into the samples when the β-CD content was 7.88 wt.% and a 0.08 and 0.04 mg mL^-1^ dexamethasone aqueous solution was used, respectively (immersion temperature: 25.0°C). When the immersion temperature was increased to 37.0°C, the proportion of dexamethasone that loaded into the hydrogels increased to ~18.16% because heat promoted the diffusion of the drug into the copolymer network and also increased the possibility of inclusion complex formation [54]. The β-CD content in the hydrogels also played a particularly important role in the amount of dexamethasone that was loaded. As the β-CD content increased, the amount of dexamethasone that was loaded also progressively increased (P<0.05). This result is potentially explained by the formation of inclusion complexes between the dexamethasone molecules and hydrophobic β-CD groups because this increases the affinity of the hydrogels for the dexamethasone molecules. Despite the changes observed in ESR and mesh size in the copolymers (as a result of the incorporation of β-CD), we were able to calculate the proportion of β-CD that was occupied by dexamethasone molecules (β-CD_IC_) as follows:

$$\beta {\text{-CD}}_{\text{IC}}\left(\%\right)=\frac{\left[{\text{m}}_{\text{pHEMA/MMA/}\beta \text{-CD}}-{\text{m}}_{\text{pHEMA/MMA}}\times \left(1{\text{-C}}_{\beta \text{-CD}}\right)\right]\times {\text{M}}_{\beta \text{-CD}}}{1000\times {\text{C}}_{\beta \text{-CD}}\times {\text{M}}_{\text{dexamethasone}}}\times 100\%$$ (3)

where m_pHEMA-MMA-β-CD_ and m_pHEMA-MMA_ are the amounts of dexamethasone (mg g^-1^) that loaded in the dried pHEMA/MMA and pHEMA/MMA/β-CD hydrogels, respectively, as shown in Table 3; M_β-CD_ and M_dexamethasone_ are the molecular weights of β-CD (1297.0 Da) and dexamethasone (392.5 Da), respectively; and C_β-CD_ is the concentration of β-CD in the pHEMA/MMA/β-CD hydrogels, as listed in Table 1.

**Table 3:** The amounts and proportions of dexamethasone that loaded into the pHEMA/MMA/β-CD hydrogels.

Sample Code	Amount of dexamethasone (mg g^-1^ of dried sample)			Percentage of dexamethasone (%)		
	^1^Immersion condition I	^2^Immersion condition II	^3^Immersion condition III	Immersion condition I	Immersion condition II	Immersion condition III
pHEMA/MMA	0.21 (0.02)	1.48 (0.19)	1.98 (0.07)	1.97 (0.66)	6.73 (1.41)	9.04 (0.31)
pHEMA/MMA/β-CD1	0.33 (0.12)	2.11 (0.15)	2.67 (0.05)	2.98 (0.25)	9.42 (1.68)	11.92 (1.18)
pHEMA/MMA/β-CD2	0.59 (0.14)	2.76 (0.15)	3.40 (0.03)	5.22 (1.36)	12.21 (0.64)	15.04 (1.47)
pHEMA/MMA/β-CD3	0.89 (0.11)	3.54 (0.28)	4.22 (0.05)	7.67 (0.22)	15.28 (1.16)	18.16 (0.98)
pHEMA/MMA/β-CD4	0.98 (0.06)	3.65 (0.16)	4.26 (0.07)	8.40 (0.56)	15.61 (0.64)	18.21 (1.29)

^1^ Immersion condition I included an immersion solution with a dexamethasone concentration of 0.04 mg ml^-1^ at 25°C;

^2^ Immersion condition II included an immersion solution with a dexamethasone concentration of 0.08 mg ml^-1^ at 25°C; and

^3^ Immersion condition III included an immersion solution with a dexamethasone concentration of 0.08 mg ml^-1^ at 37°C.

As described in Table 4, increasing the dexamethasone concentration or the immersion temperature increased the β-CD_IC_. When samples were incubated under the same immersion conditions, the β-CD_IC_ in the samples was similar despite the β-CD content. Because the total number of β-CD was higher in the hydrogels with a higher β-CD content, the amount of dexamethasone that specifically combined with β-CD was also higher. Not only can β-CD combine with dexamethasone to form inclusion complexes, but pHEMA/MMA networks can also absorb dexamethasone via non-specific binding, potentially causing the dexamethasone molecules to be hydrophobically absorbed to the hydrogel backbone, to freely diffuse within the aqueous phase of the hydrogel or to combine with water molecules via hydrogen bonds. The percentage of dexamethasone that loaded via non-specific binding (P_NC_) was estimated as follows (the variety of ESR and mesh size with the changes in β-CD content are ignored):

$${\text{P}}_{\text{NC}}\left(\%\right)=\frac{{\text{m}}_{\text{pHEMA/MMA}}\times \left({\text{1-C}}_{\beta \text{-CD}}\right)}{{\text{m}}_{\text{pHEMA/MMA/}\beta \text{-CD}}}\times 100\%$$ (4)

where m_pHEMA-MMA-β-CD_ and m_pHEMA-MMA_ are the amounts of dexamethasone (mg g^-1^) that loaded in the dried pHEMA/MMA and pHEMA/MMA/β-CD hydrogels, respectively, as shown in Table 3; and C_β-CD_ is the concentration of β-CD in the pHEMA/MMA/β-CD hydrogels, as listed in Table 1.

**Table 4:** The percentage of β-CD that was occupied by dexamethasone (β-CD_IC_) in the pHEMA/MMA/β-CD hydrogels and the percentage of dexamethasone that loaded via non-specific binding (P_NC_) out of the total loaded amount.

Sample Code	β-CD_IC_ (%)			P_NC_ (%)		
	^1^Immersion condition I	^2^Immersion condition II	^3^Immersion condition III	Immersion condition I	Immersion condition II	Immersion condition III
pHEMA/MMA	-	-	-	100 (-)	100 (-)	100 (-)
pHEMA/MMA/β-CD1	1.47 (0.23)	7.93 (0.14)	8.86 (0.23)	63.08 (1.36)	68.41 (2.41)	72.17 (0.23)
pHEMA/MMA/β-CD2	2.35 (0.12)	8.30 (0.29)	9.31 (0.17)	34.74 (2.47)	50.83 (1.38)	55.26 (1.81)
pHEMA/MMA/β-CD3	2.89 (0.21)	9.14 (0.16)	10.03 (0.33)	22.43 (0.64)	38.50 (1.73)	43.31 (2.52)
pHEMA/MMA/β-CD4	2.54 (0.14)	7.50 (0.13)	7.99 (0.24)	19.81 (1.32)	36.41 (0.85)	41.84 (0.28)

^1^ Immersion condition I included an immersion solution with a dexamethasone concentration of 0.04 mg ml^-1^ at 25°C;

^2^ Immersion condition II included an immersion solution with a dexamethasone concentration of 0.08 mg ml^-1^ at 25°C; and

^3^ Immersion condition III included an immersion solution with a dexamethasone concentration of 0.08 mg ml^-1^ at 37°C.

The P_NC_ of the samples treated with different immersion conditions are also shown in Table 4. There was a negative correlation between P_NC_ and β-CD content, whereas there were positive correlations between P_NC_ and the dexamethasone concentration and immersion temperature. This was because increasing the dexamethasone concentration or immersion temperature promoted both the migration of dexamethasone molecules from the immersion solution to the aqueous phase of the hydrogel and the formation of dexamethasone/β-CD inclusion complexes. The migration of the molecules occurred prior to the formation of the complexes. Therefore, the increase in the non-specific binding-mediated uptake of dexamethasone into the hydrogels was greater than the uptake that occurred via its specific combination with β-CD.

### Drug release *in vitro*

**Fig 6:**
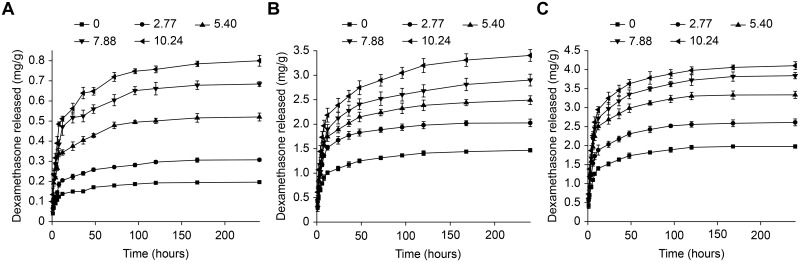
Total amount of dexamethasone released from pHEMA/MMA/β-CD hydrogels pre-soaked in dexamethasone solution (mg/g of dried hydrogels). The immersion conditions were (A) 0.04 mg mL^-1^ at 25°C, (B) 0.08 mg mL^-1^ at 25°C and (C) 0.08 mg mL^-1^ at 37°C.

IOLs that are implanted into the eyes are surrounded by aqueous humor, which is constantly circulating. Therefore, in *in vitro* assays, periodically replacing half of the released medium may simulate aqueous humor circulation. As shown in Fig 6, the *in vitro* release of dexamethasone can be sustained for at least 10 days. Its biphasic release behavior manifests as an initial burst release followed by a relatively sustained release. The release rate of dexamethasone is influenced by the following factors: (1) the diffusion of the drug from the aqueous phase, (2) the separation of drug molecules that have only weakly interacted with the hydrogel backbone, and (3) the dissociation of drug/β-CD complexes. Factors (1) and (2) are responsible for the initial burst release of dexamethasone, while factor (3) contributes to its subsequent sustained release. As described above, the P_NC_ in the samples decreased as the concentration of β-CD increased, and factor (3) therefore gradually came to replace factors (1) and (2) and to dominate the release behavior of dexamethasone.

Moreover, the higher the β-CD content in the sample is, the higher the total amount of dexamethasone that was released. We estimated that the cumulative dexamethasone release rates over the first 24 h in the samples with β-CD contents of 0 wt.% and 10.24 wt.% were ~71.3% and ~57.3%, respectively (immersion condition: 0.04 mg mL^-1^ at 25°C). Therefore, adding β-CD to the hydrogels slowed the rate of dexamethasone release.

In addition, we found that the total amount of dexamethasone released during the initial 2–3 days (burst release stage) was higher than the amount that dissolved in the aqueous phase and combined with the hydrogel backbone. These results indicate that the dissociation of a portion of the drug/β-CD inclusion complexes occurred in the initial stage. Two things should be noted here. First, the burst release of the drug may have promoted the dissociation of the inclusion complexes. Second, the fact that we periodically replaced half of the release medium may also have enhanced the dissociation.

When samples were immersed in the lower concentration of dexamethasone (0.04 mg mL^-1^), the hydrogels become loaded with a lower amount of dexamethasone, and the P_NC_ was smaller. Therefore, a relatively weaker burst release and a relatively slower sustained release were observed. In contrast, heating the drug during the loading stage resulted in a relatively stronger burst release of dexamethasone. Nevertheless, a higher dexamethasone concentration was observed in the samples immersed in an aqueous solution and those that were heated. Both of these treatments may have increased the total amount of dexamethasone that was released because under these conditions, the hydrogels can load more dexamethasone.

For patients who undergo cataract surgery, the incidence and seriousness of post-operative inflammation are much higher during the earlier recovery time than during the following stages [55]. The results of this study show that pHEMA/MMA/β-CD hydrogels loaded with dexamethasone may benefit these patients by controlling post-operative inflammation because the biphasic release behavior of dexamethasone corresponds relatively well to the regular pattern of occurrence of post-operative inflammation.

### Cytotoxicity

**Fig 7:**
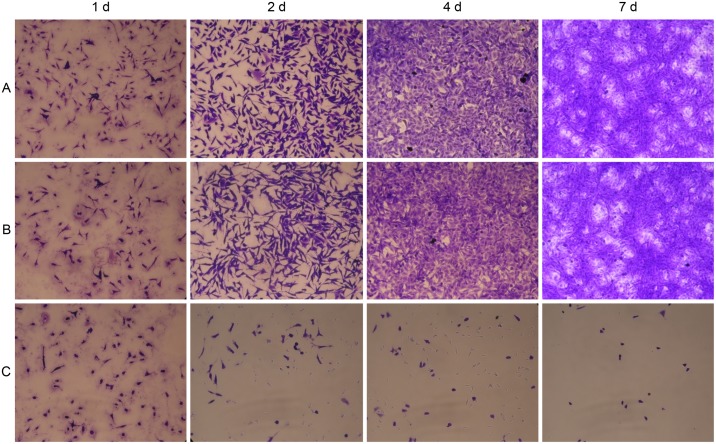
Inverted phase contrast microscopy images of the morphologies of L929 mouse connective tissue fibroblasts. (A) HDPE: negative controls, (B) pHEMA/MMA/β-CD3 hydrogels with 7.88 wt.% β-CD, and (C) PVC: positive controls. All wet samples are stained by Using Crystal Violet and all microscopy images are shown at the same magnification (40×).

The cytotoxicity of the sterilized hydrogels was analyzed using L929 fibroblasts. The L929 cells were incubated in direct contact with the samples for 1, 2, 4 and 7 days. The results shown in Fig 7 demonstrate the influence of the samples on cell morphology, which was assessed using crystal violet staining. L929 cells that were cocultured with pHEMA/MMA/β-CD hydrogels are compared to the positive controls (toxic organotin PVC) and the negative controls (HDPE) in images obtained using phase contrast microscopy. As shown, the cells incubated with HDPE (negative control) and normal L929 fibroblasts were fusiform- or star-shaped cells that proliferated and formed an aggregated monolayer as the incubation time increased. In contrast, normal nuclear and cytoplasmic morphologies were rarely observed in the L929 cells cocultured with organotin PVC (positive control). Additionally, the view fields are full of residual granular or cord-like cell debris, indicating cell death and cell lysis. The L929 cells incubated with pHEMA/MMA/β-CD3 hydrogels maintained a favorable polygonal shape with stretched filopodia, which is a typical morphology of normal L929 cells. No detachment and no debris were observed in these cocultures.

**Fig 8:**
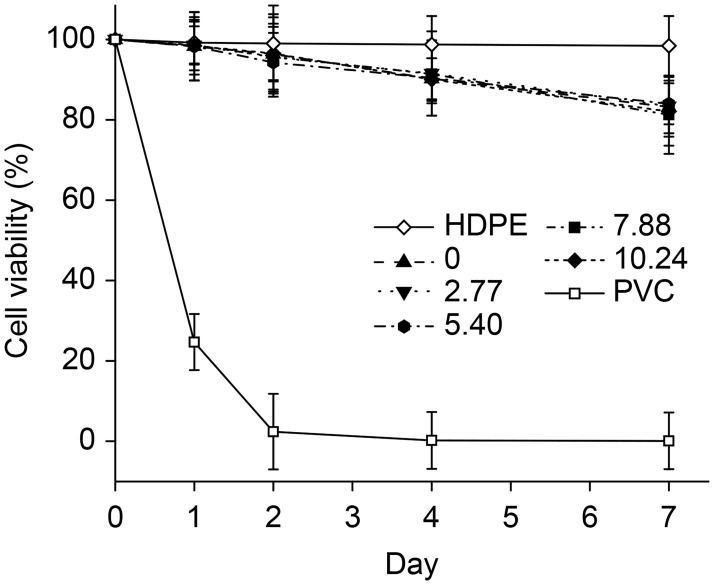
Cell viability in L929 mouse connective tissue fibroblasts of pHEMA/MMA/β-CD hydrogels assessed using MTT assays.

Fig 8 shows the results of MTT assays that were performed to quantitatively characterize viability and proliferation in cell cultures. The values obtained for cell viability in the L929 cells were similar among the groups of pHEMA/MMA/β-CD hydrogels and the negative control on day 1, whereas in the positive control, cell viability decreased to ~25.0% on day 1 and was nearly 0% on day 2. After 7 days of co-culture, the cell viability rate remained ~100.0% in the negative control but was slightly lower in the pHEMA/MMA/β-CD hydrogels groups. These results indicate that hydrogels used in this study exhibited good biocompatibility.

## Conclusions

In this study, we successfully prepared and characterized pHEMA/MMA/β-CD hydrogels that exhibit a sustained release pattern when loaded with dexamethasone. The optical properties, thermomechanical properties, and ESR of the hydrogels can be adjusted by changing the β-CD content. A higher β-CD content in the hydrogels was associated with a higher concentration of loaded dexamethasone. Additionally, introducing β-CD extended the drug-releasing time. Moreover, heating the drug-containing solution increased the amount of dexamethasone that was loaded and subsequently released by the hydrogels. In addition, increasing the β-CD content increased the hydrophilicity of the hydrogels. The water CA was consequently lower, and this alteration was accompanied by an increase in the number of LECs that adhered to the surface of the hydrogels. The hydrogels showed good biocompatibility with L929 mouse connective tissue fibroblasts. These *in vitro* results inspired us to perform *in vivo* experiments aimed at testing the anti-inflammatory effects of drug-loaded, sustained-release IOLs based on the pHEMA/MMA/β-CD hydrogels presented in this study, and these results will be reported in a later publication. In addition, previous studies demonstrated that β-CD can encapsulate a variety of small molecule drugs, including antibiotics and anti-glaucoma drugs [56,57]. Therefore, IOLs composed of pHEMA/MMA/β-CD hydrogels may be useful for treating complicated cataracts, including uveitis cataract and glaucoma with cataract.

## Supporting information

S1 Fig Illustration of pHEMA/MMA/β-CD IOLs with the capability of maintaining the sustained release of anti-inflammatory drugs. (PDF)

S2 Fig The synthesis process of triple-MA-β-CD. (PDF)

S3 Fig Weight loss ratio of the pHEMA/MMA/β-CD hydrogels as a consequence of β-CD content. (PDF)

S4 Fig Weight of the pHEMA/MMA/β-CD disks as a consequence of β-CD content. (PDF)
