# Supplementary material for: Cyclodextrin-containing hydrogels as an intraocular lens for sustained drug release
Source: PLoS One. 2017 Dec 15;12(12):e0189778. doi: 10.1371/journal.pone.0189778 (PMC5731761; doi:10.1371/journal.pone.0189778)
Supplement: S1 Fig — (PDF) [file pone.0189778.s001.pdf]

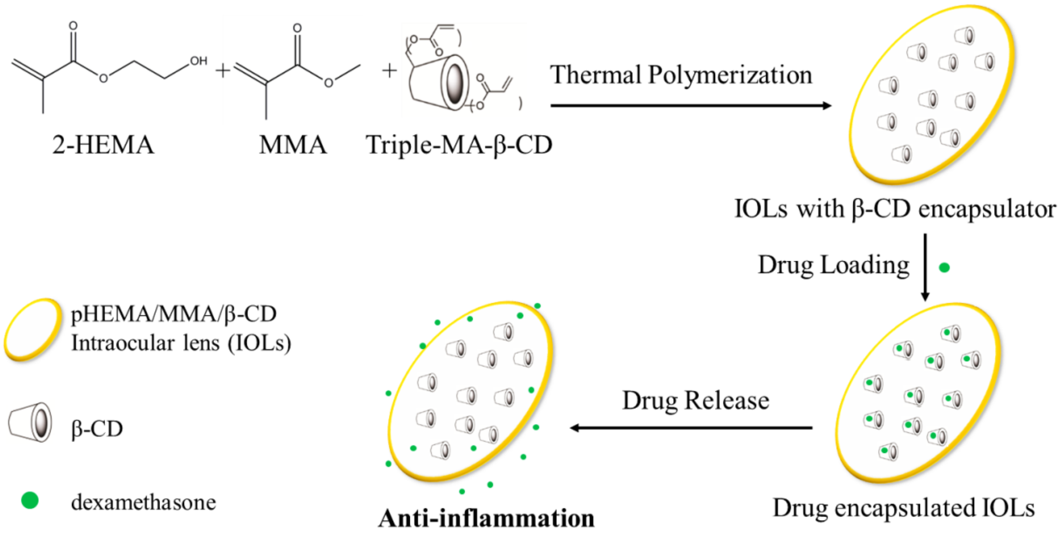

S1 Fig. Illustration of pHEMA/MMA/β-CD IOLs with the Capability of Maintaining the Sustained Release of Anti-Inflammatory Drugs.
