# Supplementary figures and images for: Cyclodextrin-containing hydrogels as an intraocular lens for sustained drug release
Source: PLoS One. 2017 Dec 15;12(12):e0189778. doi: 10.1371/journal.pone.0189778 (PMC5731761; doi:10.1371/journal.pone.0189778)

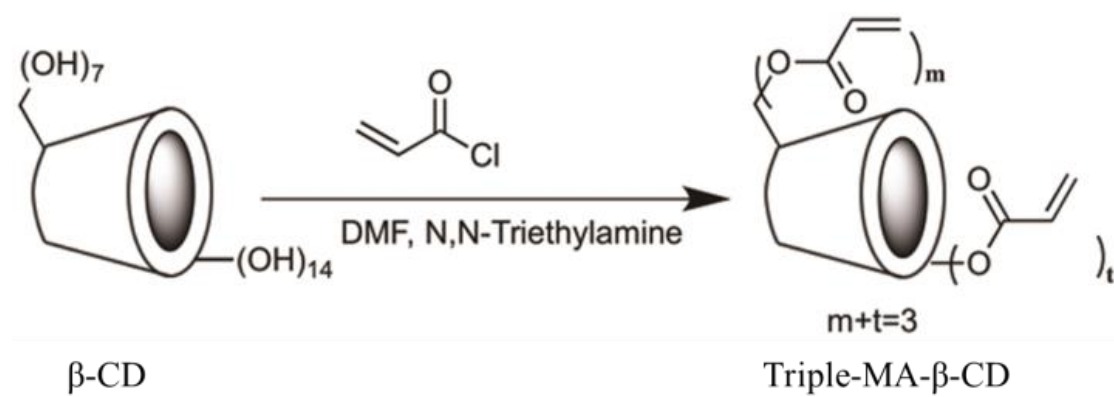

S2 Fig. The synthesis process of Triple-MA- $\beta$ -CD

Supplement: S2 Fig — (PDF) [file pone.0189778.s002.pdf]

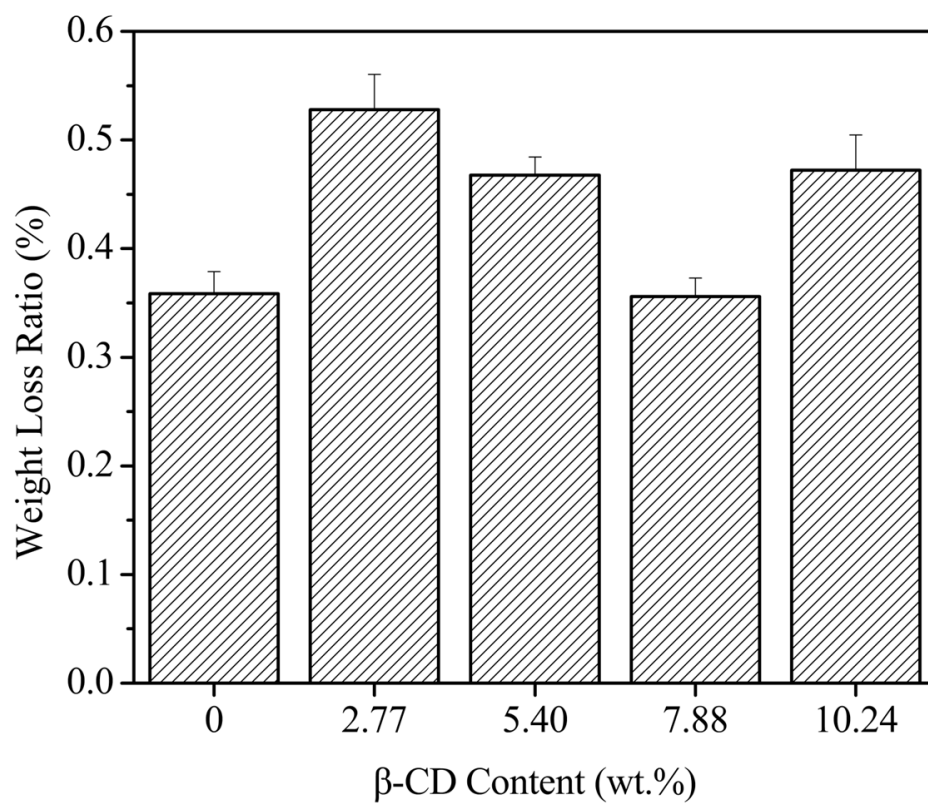

S3 Fig. Weight loss ratio of the pHEMA/MMA/β-CD hydrogels as a consequence of β-CD content

Supplement: S3 Fig — (PDF) [file pone.0189778.s003.pdf]

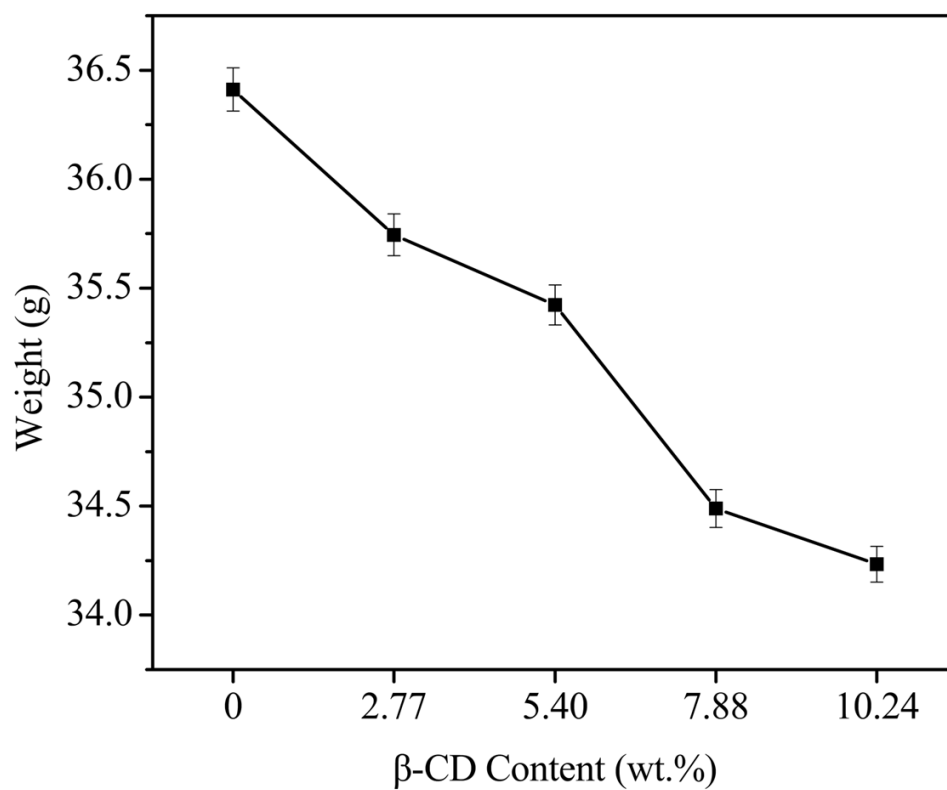

S4 Fig. Weight of the pHEMA/MMA/β-CD disks as a consequence of β-CD content

Supplement: S4 Fig — (PDF) [file pone.0189778.s004.pdf]
